# Urgency Prediction for Medical Laboratory Tests Through Optimal Sparse Decision Tree: Case Study With Echocardiograms

**DOI:** 10.2196/64188

**Published:** 2025-01-29

**Authors:** Yiqun Jiang, Qing Li, Yu-Li Huang, Wenli Zhang

**Affiliations:** 1 Robert D. and Patricia E. Kern Center for the Science of Health Care Delivery Mayo Clinic Rochester, MN United States; 2 Department of Industrial & Manufacturing Systems Engineering Iowa State University Ames, IA United States; 3 Department of Information Systems and Business Analytics Iowa State University Ames, IA United States

**Keywords:** interpretable machine learning, urgency prediction, appointment scheduling, echocardiogram, health care management

## Abstract

**Background:**

In the contemporary realm of health care, laboratory tests stand as cornerstone components, driving the advancement of precision medicine. These tests offer intricate insights into a variety of medical conditions, thereby facilitating diagnosis, prognosis, and treatments. However, the accessibility of certain tests is hindered by factors such as high costs, a shortage of specialized personnel, or geographic disparities, posing obstacles to achieving equitable health care. For example, an echocardiogram is a type of laboratory test that is extremely important and not easily accessible. The increasing demand for echocardiograms underscores the imperative for more efficient scheduling protocols. Despite this pressing need, limited research has been conducted in this area.

**Objective:**

The study aims to develop an interpretable machine learning model for determining the urgency of patients requiring echocardiograms, thereby aiding in the prioritization of scheduling procedures. Furthermore, this study aims to glean insights into the pivotal attributes influencing the prioritization of echocardiogram appointments, leveraging the high interpretability of the machine learning model.

**Methods:**

Empirical and predictive analyses have been conducted to assess the urgency of patients based on a large real-world echocardiogram appointment dataset (ie, 34,293 appointments) sourced from electronic health records encompassing administrative information, referral diagnosis, and underlying patient conditions. We used a state-of-the-art interpretable machine learning algorithm, the optimal sparse decision tree (OSDT), renowned for its high accuracy and interpretability, to investigate the attributes pertinent to echocardiogram appointments.

**Results:**

The method demonstrated satisfactory performance (*F*_1_-score=36.18% with an improvement of 1.7% and *F*_2_-score=28.18% with an improvement of 0.79% by the best-performing baseline model) in comparison to the best-performing baseline model. Moreover, due to its high interpretability, the results provide valuable medical insights regarding the identification of urgent patients for tests through the extraction of decision rules from the OSDT model.

**Conclusions:**

The method demonstrated state-of-the-art predictive performance, affirming its effectiveness. Furthermore, we validate the decision rules derived from the OSDT model by comparing them with established medical knowledge. These interpretable results (eg, attribute importance and decision rules from the OSDT model) underscore the potential of our approach in prioritizing patient urgency for echocardiogram appointments and can be extended to prioritize other laboratory test appointments using electronic health record data.

## Introduction

### Background

In the present medical landscape, the intricate interplay between innovative techniques has expanded the horizons of medical knowledge and opened avenues for unprecedented precision in patient care. The increasingly sophisticated laboratory tests play a crucial role in this transformative process. Born out of meticulous research and honed by the rigors of scientific scrutiny, these tests provide clinicians with a multifaceted toolkit to decipher the intricacies of illnesses, capturing the nuances of each condition, guiding medical professionals toward evidence-based interventions, and empowering medical professionals to tailor treatments with personalized precision.

However, a pivotal factor to take into consideration is the limited availability of certain state-of-the-art laboratory tests, as they often involve intricate equipment and elaborate protocols. This is evident from their expensive nature, the scarcity of skilled medical professionals capable of operating these laboratories, and the limited accessibility across different regions or during specific time frames [1]. As a result, the transformative potential of these laboratory tests is mitigated by the practical challenges they pose in terms of affordability [2]. The potential significant advantages of laboratory tests, coupled with their limited availability, render them a scarce resource, resulting in many patients having to endure wait times for access to laboratory tests. Consequently, predicting and prioritizing which patients require testing has emerged as an important research problem.

The rise of health IT and the subsequent influx of electronic health record (EHR) data, combined with the power of machine learning, offers new opportunities to revolutionize the prioritization of medical laboratory tests [3]. By delving into vast amounts of historical patient information, machine learning algorithms can discern intricate patterns and correlations that might otherwise elude human observation. The predictive outcomes generated by machine learning algorithms can contribute to refining testing protocols, enabling medical practitioners to make data-driven decisions regarding the prioritization and scheduling of laboratory tests based on patient information. In this study, we aim to elucidate methods for evaluating patients’ urgency for tests, seeking to refine the allocation of scarce laboratory tests by harnessing the power of machine learning and analyzing historical EHRs. Specifically, we aim to contribute by applying an optimal sparse decision tree (OSDT) to a new domain—predicting the urgency of medical laboratory tests, using echocardiograms as a case study. Based on our literature review, OSDT stands out as one of the most suitable methods for achieving both optimal performance and interpretability in predicting the urgency of patients requiring echocardiograms. Our ultimate objective is to ensure prompt access for patients with the most critical needs.

### Related Work

#### Echocardiogram and Patient Prioritization Techniques

An echocardiogram is one the most cost-effective means for screening cardiac anatomy, uses ultrasound to evaluate the cardiac structures, and provides critical information for medical providers [4]. It functions as a crucial precursor to a detailed diagnosis, capable of screening cardiac anatomy and providing essential information for assessing cardiovascular conditions such as murmurs, stenosis, and regurgitation. Additionally, it plays a crucial role in diagnosing valvular morphology and uncovering the root causes of valve diseases [5]. A comprehensive echocardiographic assessment can provide both diagnostic and prognostic information, thus facilitating risk stratification and establishing baseline data for future evaluations [5].

The echocardiogram, although immensely valuable, is not always easily attainable due to the increasing demand for the test. For example, there has been an observed increase in the prevalence of rheumatic heart disease, which stands as the most predominant form of valvular heart disease and impacts approximately 41 million individuals in developing countries [6]. In recent years, there has been a notable escalation in the demand for pediatric cardiology services, leading to documented workloads that have exhibited a substantial upsurge of up to 51% over the past decades [7]. Furthermore, there has been an increase in the prevalence of children with asymptomatic murmurs who necessitate evaluation through echocardiogram [8]. The increasing demands pose challenges to echocardiogram laboratories in resource management, requiring medical institutions to establish more effective scheduling protocols to prioritize patients in critical need of echocardiogram lab appointments.

Patient prioritization techniques can be broadly classified into scoring systems and machine learning classification–based systems [9]. Scoring systems, particularly those using regression techniques, have gained prominence for their ability to allocate medical resources. These systems heavily rely on the expertise of medical professionals to assign priority scores to patients. Examples include the Salisbury priority scoring system, allowing surgeons to assign relative priorities, and the Italian waiting time prioritization system, which reallocates outpatient referrals based on clinical priorities prescribed by general practitioners [9]. These methods, however, exhibit various limitations. First, there may be inherent bias (eg, subjective judgments obtained through experience by medical professionals) as these approaches often necessitate input from medical specialists’ judgments. A machine learning and data-driven method can serve as a complement to these types of systems. Second, these methods might be tailored for a particular patient prioritization task (eg, surgery or referral), and demand a high level of specialized medical knowledge for their design, making them difficult to generalize to other tasks [10]. Third, certain methods lack transparent decision rules for assessing the significance of input attributes, thereby posing challenges for their practical applications [11]. Machine learning classification-based methods typically rely on a large amount of patients’ information (eg, EHRs) to autonomously discern patterns and generate predictions. This process aids in patient prioritization and avoids limitations associated with scoring systems [12]. The existing methods, however, fail to transform the prediction process and outcomes into clear and executable rules, limiting the practical application of these approaches [9]. Moreover, existing studies predominantly center around 5 clinical areas, including cataract surgery, general surgical procedures, hip and knee replacements, magnetic resonance imaging scanning, and children’s mental health using specific predictive attributes and expert systems [13]. There is a crucial need for new methods that apply more broadly to general laboratory test prioritization.

To summarize, our literature review underscores the need for new methods of prioritizing patients, which leverage machine learning and data-driven techniques to complement existing methods, ensure transparency, and have the potential to be generalized to various patient prioritization tasks. Consequently, using extensive patient historical EHRs combined with an interpretable machine learning approach emerges as a potential solution to address these gaps.

#### Leveraging Machine Learning for Optimizing the Use of Scarce Laboratories Tests

When a large number of patient EHRs, which contain numerous hidden patterns, are available, integrating machine learning into health care practices emerges as a potential solution to address pressing issues such as the continual demand for medical services outpacing available resources. Specifically, machine learning, with its capacity to analyze vast data and discern intricate patterns, empowers health care professionals to make data-driven decisions regarding the allocation of laboratory tests. By developing predictive models using historical EHRs, machine learning models can identify individuals who are more likely to benefit from specific tests, ensuring that scarce resources are allocated where they can yield the greatest impact. Furthermore, such methods ensure critical cases receive prompt attention, leading to expedited diagnoses and interventions [14]. Moreover, the prediction results can potentially streamline the testing process by reducing unnecessary tests [15].

The integration of machine learning techniques to optimize the allocation of limited medical tests and laboratory resources has attracted considerable research attention. Research by Elitzur et al [16] delves into the use of prediction models to allocate medical tests efficiently. The study uses historical patient data to develop models that identify the most suitable candidates for specific tests, thereby enhancing resource allocation and streamlining the testing process. In a similar vein, Marescotti et al [17] investigate the orchestration of laboratory workflows through machine learning-driven prioritization. By considering factors such as clinical urgency and resource availability, their work demonstrates how machine learning algorithms can ensure timely and effective laboratory test processing, contributing to both improved patient care and optimized resource use. Similarly, Zhang et al [18] estimate the probability of requiring mechanical ventilation for in-hospital patients and contribute to the literature by identifying which patients require medical devices (ie, critical medical resources) more urgently.

However, while the potential benefits of machine learning in optimizing resource allocation are evident, challenges remain. A recent study underscores the need for further research and development in the area of machine learning models’ interpretability and fairness, ensuring that data-driven decisions in health care maintain transparency [19]. The research gap drives us to use an interpretable and efficient machine learning method for laboratory tests and patient optimization.

#### Interpretable Machine Learning

Medical research is often at the forefront of technological innovation, with machine learning algorithms being harnessed to analyze vast datasets, predict disease outcomes, and assist in clinical decision-making. However, as these algorithms become increasingly sophisticated, they tend to function as “black boxes,” where the reasoning behind their predictions remains obscured. This opacity not only raises concerns about trustworthiness but also impedes the adoption and acceptance of these tools by medical professionals [19].

In medical research, the concept of interpretability holds profound significance. The intricate interplay between cutting-edge technology and human well-being underscores the critical need to not only generate accurate predictions but also to understand the underlying rationale behind those predictions. The complexity of medical data, coupled with the potential life-altering consequences of decisions made based on data and machine learning models, demands a heightened level of transparency and comprehensibility requirements [20].

The interpretability of machine learning models empowers health care providers to understand the factors that led to a specific decision, enabling them to fine-tune treatment strategies according to their medical judgment and the patient’s unique circumstances. Consequently, there has been a surge in post hoc techniques for elucidating black box machine learning models in a manner interpretable by humans. The most prominent techniques among these include local, model-agnostic methods that aim to explain individual predictions of a given black box classifier, such as local interpretable model-agnostic Explanation and Shapley additive explanation [21]. Due to their high generalizability, post hoc methods have been used to explain a wide array of machine learning models across various domains. However, previous research has indicated that there are common limitations associated with these post hoc techniques, including local interpretability, sensitivity to perturbations, and difficulties in choosing interpretable surrogate models [21].

In health care, arguably, a more appropriate research direction for using interpretable machine learning is tree-based models because much of the data related to patient prioritization is structured data (eg, tabular EHRs). Tree-based machine learning models can perform comparably to complex models (eg, deep learning models), especially after thorough preprocessing of tabular data [22]. In contrast to post hoc explainable machine learning techniques, tree-based models are logical models that consist of statements involving logical operations, providing clear and interpretable decision rules [22]. This interpretability is highly valuable in health care, as it allows medical professionals to not only make accurate predictions but also understand the underlying factors driving those predictions, enhancing transparency and trust in the decision-making process.

Since our research aims to use historical EHR data for patient prioritization, it is crucial to acknowledge another notable characteristic of patient prioritization-related information: the prevalence of numerous categorical variables (eg, patient demographic information such as gender and age groups). Furthermore, the outcomes of patient prioritization are also expressed as categorical variables. For example, preventive interventions often involve categorical decisions, such as determining which individuals should undergo selective or indicated interventions or identifying those most likely to benefit from specific treatments [23]. In such scenarios, an efficient tree-based approach tailored to categorical variables is highly valuable. In this study, we focus on a cutting-edge decision tree algorithm–OSDT [24].

A decision tree features a hierarchical structure that is composed of a root node, branches, internal nodes, and leaf nodes in a tree format. Each path from the root node to the leaf node illustrates a rule to partition the data and leads to the final classification. The tree-based method presents a clear pattern for the decision-making process; thus, it is considered a transparent and highly interpretable model [25]. The results of the tree-based models are extremely useful for medical decision-making [26], and the performance of decision tree classifiers is verified by researchers on medical data [27]. Nevertheless, concerns have been raised regarding the suboptimality of decision tree algorithms [24,28]. To address this issue, OSDT has been introduced, aiming to ensure optimal solutions for binary variables in a computationally efficient manner [24].

The OSDT algorithm addresses various limitations observed in prior tree-based methods. Unlike previous approaches that often focused on finding the optimal tree within a fixed number of nodes or limited topology, OSDT tackles these shortcomings by identifying optimal trees through the use of a regularized loss function. This loss function strikes a balance between accuracy and the number of leaves, thereby enhancing the efficiency of the decision tree model. Furthermore, OSDT improves computational efficiency and interpretability by incorporating a series of analytical bounds that effectively reduce the search space while still identifying the optimal tree. By implementing these bounds, the algorithm streamlines the search process, leading to expedited identification of the optimal decision tree structure. Moreover, the OSDT algorithm has undergone mathematical validation, demonstrating its efficacy in constructing optimal trees for structured tabular datasets with attributes having binary values. It establishes its effectiveness in addressing binary classification problems. The algorithm is designed to uphold commendable levels of accuracy and is anticipated to meet the demands of medical prediction tasks with stringent interpretability requirements.

## Methods

### Study Design

In this study, we conducted empirical and predictive analyses using echocardiogram data extracted from EHRs at a large multispecialty hospital and medical facility. The dataset included administrative details, referral diagnoses, and patient conditions. To explore attributes relevant to echocardiogram prioritization, we used the OSDT algorithm due to its high accuracy and interpretability. We aim to enhance the scheduling of echocardiogram laboratory appointments by enabling the prioritization of patients with urgent needs based on our model’s predictions. To be noted, our proposed method is not intended to replace human expertise but to complement it, offering valuable insights that guide practitioners toward informed and patient-centric choices.

### Ethical Considerations

The Mayo Clinic Institutional Review Board, based on the authors' submission notes and in accordance with the Code of Federal Regulations, 45 CFR 46.102, deemed that this research did not require IRB review.

### Data Collection and Selection

The dataset comprises real-world data from one of the top medical centers in the United States. The data were collected over a 1-year period in 2019, including 34,293 echocardiogram appointments. It consisted of 64 dummy-coded categorical attributes, encompassing various aspects such as patient demographics, medical history, clinical settings (eg, inpatient or outpatient status), past procedures, future scheduled procedures, and diagnose indicators for echocardiogram-justifying signs (eg, heart murmurs, shortness of breath, or chest pain) extracted from the clinical notes and referrals in the EHRs (Table 1).

The dataset exhibited a notable class imbalance issue, particularly evident in the examination of the “MadeBeforeEcho” attribute. This attribute delineates whether the downstream appointment following the echocardiogram occurs before the scheduling date of the echocardiogram appointment (not the actual appointment date). Within the “Y” category, the distribution revealed 84% nonurgent cases and 16% urgent cases. Conversely, in the “N” category, the distribution portrayed 58% nonurgent cases and 42% urgent cases. This observation underscored a substantial prevalence of nonurgent cases within the “MadeBeforeEcho” attribute. Furthermore, a similar pattern of imbalance is discerned when analyzing attributes such as “ReferredType” and “SurgeryYN.” These attributes also exhibit a significant majority of cases concentrated within 1 category, indicating the need for careful consideration of class distribution in subsequent predictions.

The response variable is determined by calculating the number of days between the date the echocardiogram appointment was generated in the system and the actual appointment date. According to medical policy, appointments are classified as urgent (ie, the response variable) if the number of days is 2 or less, and nonurgent otherwise.

**Figure 1:**
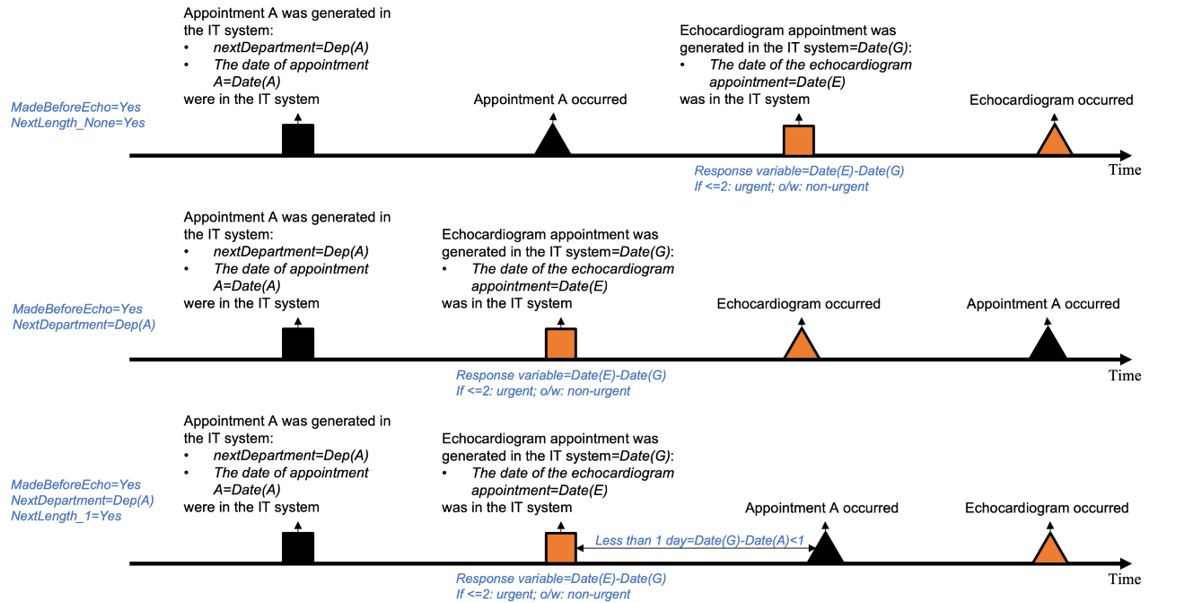
Timeline and process of echocardiogram appointment scheduling. Using MadeBeforeEcho as an example.

It is important to note that the features categorized under the “Future Scheduled Process” were derived based on the date the echocardiogram appointment is generated in the system, rather than the actual appointment date (Figure 1). This approach ensures that the model uses only the information available up to the point of echocardiogram appointment generation, without incorporating any data beyond this cutoff.

Of note, our dataset is a tabular dataset with attributes and response variables having binary values. Therefore, OSDT is highly suitable for serving this dataset, assisting us in making predictions for patient prioritization.

**Table 1:** Dataset and attribute statistics^a^.

Category and variable				Description			Summary statistics, n (%)			
							Nonurgent			Urgent
**Demographics**										
	**Age (years)**									
		0-18	—^b^		1929 (7.18)			478 (6.41)		
		19-55	—		6766 (25.19)			1930 (25.90)		
		56-65	—		4954 (18.45)			1342 (18.01)		
		66-75	—		6784 (25.26)			1896 (25.44)		
		Older than 75	—		6398 (23.82)			1775 (23.82)		
	**Sex**									
		Female	—		11,829 (44.09)			3529 (47.55)		
		Male	—		15,002 (55.91)			3892 (52.45)		
	**Patient geolocation**									
		In_State	—		9973 (37.14)			2376 (31.96)		
		Out_of_State	—		14,332 (53.37)			4301 (57.85)		
		Town	—		2550 (9.50)			758 (10.20)		
**Clinical settings**										
	**ReferralType**									
		External	—		1156 (4.30)			606 (8.15)		
		Internal	—		25,699 (95.70)			6829 (91.85)		
	**ReferredBy**		The specialty that patient referred by							
		Cardiovascular medicine	—		8188 (30.49)			1162 (15.63)		
		Family medicine	—		436 (1.62)			142 (1.91)		
		Hospital medicine	—		145 (0.54)			4 (0.05)		
		Internal medicine	—		978 (3.64)			591 (7.95)		
		Obstetrics and gynecology	—		1096 (4.08)			359 (4.83)		
		Pediatric and adolescent medicine	—		2302 (8.57)			401 (5.39)		
		Other	—		13,710 (51.05)			4776 (64.24)		
	**ReferredFrom**		Referral origin							
		Arizona campus	—		2 (0.01)			0 (0.00)		
		Florida campus	—		1 (0.00)			0 (0.00)		
		Mayo Clinic health system	—		154 (0.57)			38 (0.51)		
		Rochester campus	—		17,495 (65.15)			4463 (60.03)		
		Other	—		9203 (34.27)			2934 (39.46)		
	**ReferredType**		Referred type							
		Outpatient	—		18,706 (69.66)			4585 (61.52)		
		Other	—		8149 (30.34)			2868 (38.48)		
**Future scheduled process**										
	**Diff_surgery_after**		The number of days between the date the echocardiogram appointment was generated in the system and the surgery date							
		0-1	—		1449 (5.40)			461 (6.20)		
		2-5	—		1607 (5.98)			492 (6.62)		
		6-15	—		1143 (4.26)			606 (8.15)		
		16 and greater	—		4715 (17.56)			1494 (20.09)		
		None	—		17,941 (66.81)			4382 (58.94)		
	**MadeBeforeEcho**		Whether the next downstream appointment after echocardiogram is made before the date the echocardiogram appointment was generated in the system or not							
		Yes	—		23,845 (88.79)			4660 (62.53)		
		No	—		3010 (11.21)			2793 (37.47)		
	**NextDepartment**		The department in which the appointment happened after the date the echocardiogram appointment was generated in the system							
		Cardiovascular medicine	—		12,012 (44.73)			1749 (23.47)		
		Non-cardiovascular medicine	Departments other than cardiovascular medicine		14,843 (55.27)			5704 (76.53)		
	**NextLength**		The number of days from the date the echocardiogram appointment was generated in the system to its following appointment							
		0-1	—		4531 (16.87)			1608 (21.63)		
		1-5	—		3301 (12.29)			2018 (27.14)		
		Greater than 5	—		1,014 (3.78)			618 (8.31)		
		None	—		18,009 (67.06)			3191 (42.92)		
	**Procedure**		Type of echocardiogram visit							
		TEE^c^	—		848 (3.16)			362 (4.87)		
		TTE^d^	—		23,293 (86.74)			6803 (91.50)		
		Other	—		2714 (10.11)			270 (3.63)		
**Past procedures**										
	**SurgeryYN**		Whether the patient had a cardiovascular surgery within 6 months prior to the date the echocardiogram appointment was generated in the system							
		Yes	—		1708 (6.36)			264 (3.54)		
		No	—		25,147 (93.64)			7189 (96.46)		
	**SurgeryYN_After**		Whether the patient had a surgery within 3 months after the date the echocardiogram appointment was generated in the system							
		Yes	—		8914 (33.19)			3053 (40.96)		
		No	—		17,941 (66.81)			4400 (59.04)		
**Medical history**										
	Alcohol		Alcohol abuse			115 (0.43)			50 (0.67)	
	Anemia		Anemia			962 (3.58)			605 (8.12)	
	BloodLoss		Blood loss			87 (0.32)			33 (0.44)	
	CHF^e^		—			1884 (7.02)			484 (6.49)	
	Coagulopathy		Coagulation deficiency			446 (1.66)			274 (3.68)	
	Depression		Major depressive disorder			439 (1.63)			192 (2.58)	
	DM^f^		—			610 (2.27)			230 (3.09)	
	DMcx^g^		—			317 (1.18)			129 (1.73)	
	Drugs		Drug abuse			86 (0.32)			19 (0.25)	
	FluidsLytes		Fluid and electrolyte disorders			1013 (3.77)			617 (8.28)	
	HIV		—			0 (0.00)			1 (0.01)	
	Hypertension		—			2201 (8.20)			786 (10.55)	
	Hypothyroid		Hypothyroidism			777 (2.89)			277 (3.72)	
	Liver		—			429 (1.60)			197 (2.64)	
	Lymphoma		Lymph system cancer			464 (1.73)			347 (4.66)	
	Metastatic cancer		—			251 (0.93)			222 (2.98)	
	NeuroOther		Neurological disorders			581 (2.16)			291 (3.90)	
	Obesity		—			980 (3.65)			339 (4.55)	
	Paralysis		—			58 (0.22)			15 (0.20)	
	PHTN^h^		Pulmonary circulation disorders			298 (1.11)			153 (2.05)	
	Psychoses		Mental disorder characterized by a disconnection from reality			126 (0.47)			53 (0.71)	
	PUD^i^		Chronic peptic ulcer			41 (0.15)			20 (0.27)	
	Pulmonary		Chronic pulmonary disease			650 (2.42)			273 (3.66)	
	PVD^j^		—			965 (3.59)			234 (3.14)	
	Renal		Renal failure			950 (3.54)			331 (4.44)	
	Rheumatic		Rheumatoid arthritis or collagen vascular			254 (0.95)			150 (2.01)	
	Tumor		Solid tumor			722 (2.69)			380 (5.10)	
	Valvular		Valvular disease			3367 (12.54)			573 (7.69)	
	WeightLoss		Weight loss			248 (0.92)			237 (3.18)	
**Diagnoses**										
	A		MSSA^k^ bacteremia, sepsis			18 (0.07)			25 (0.34)	
	B		MRSA^l^, staph bacteremia, slaph, fungemia, pseudomonas, candidemia, MRSA bacteremia			47 (0.18)			40 (0.54)	
	C		Leukemia, AML^m^, CML^n^, lymphoma, AMV^o^, myeloma			1428 (5.32)			554 (7.43)	
	D		Diseases of the blood and blood-forming organs and certain disorders involving the immune mechanism			561 (2.09)			193 (2.59)	
	E		Endocrine, nutritional and metabolic diseases			1714 (6.38)			408 (5.74)	
	F		Behavioral and neurodevelopmental disorders			49 (0.18)			46 (0.62)	
	G		Muscular dystrophy			590 (2.20)			273 (3.66)	
	H		Diseases of the eye and adnexa or disease of the ear and mastoid process			60 (0.22)			28 (0.38)	
	I		Heart failure, coronary artery, cardiac arrest, STEMI^p^, stroke, cardia, hypertension, endocarditis, NSTEMI^q^, PEA^r^ arrest, AFib^s^, pulmonary embolism, pulmonary hypertension, and vegetation			11,302 (42.09)			4096 (54.96)	
	J		Resp failure, respiratory, and pulmonary			477 (1.78)			392 (5.26)	
	K		Liver and cirrhosis			357 (1.33)			130 (1.74)	
	L		Diseases of the skin and subcutaneous tissue			36 (0.13)			33 (0.44)	
	M		Diseases of the musculoskeletal system and connective tissue			503 (1.87)			280 (3.76)	
	N		Diseases of the genitourinary system			397 (1.48)			119 (1.60)	
	O		Pre-eclampsia, preeclampsia			235 (0.88)			57 (0.76)	
	P		Certain conditions originating in the perinatal period			12 (0.04)			4 (0.05)	
	Q		Ehlers, coarc, PDA^t^, and congenital			2811 (10.47)			309 (4.15)	
	R		Murmur, hypoxemia, shortness, SOB^u^, breath, shock, dyspnea, chest pain, troponin, syncope, electrocardiogram, extremity, mass, and swelling, edema			4111 (15.31)			2811 (37.72)	
	S		Injury, poisoning and certain other consequences of external causes			100 (0.37)			21 (0.28)	
	Z		Chemo, preoperative, pre-op, prenatal, pregnancy, prior to, BMI, surgery, and transplant			5966 (22.22)			1129 (15.15)	

^a^All the features used in this study are complete for each patient, with no missing values. The diagnoses are derived from patients’ ICD-9 codes, and the medical history is extracted from electronic health record notes using the medical center’s built-in natural language processing tools.

^b^Not applicable.

^c^TEE: transesophageal echocardiogram.

^d^TTE: transthoracic echocardiogram.

^e^CHF: congestive heart failure.

^f^DM: diabetes without chronic complications.

^g^DMcx: diabetes with chronic complications.

^h^PHTN: pulmonary hypertension.

^i^PUD: peptic ulcer disease.

^j^PVD: peripheral vascular disease.

^k^MSSA: methicillin-sensitive *Staphylococcus aureus*.

^l^MRSA: methicillin-resistant *Staphylococcus aureus*.

^m^AML: acute myeloid leukemia.

^n^CML: chronic myeloid leukemia.

^o^AMV: avian myeloblastosis virus.

^p^STEMI: ST-elevation myocardial infarction.

^q^NSTEMI: non–ST-elevation myocardial infarction.

^r^PEA: pulseless electrical activity.

^s^AFib: atrial fibrillation.

^t^PDA: patent ductus arteriosus.

^u^SOB: shortness of breath.

### Problem Formulation: Urgency Prediction Using OSDT

With data 
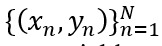
 , where 
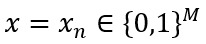
 are *M* binary attributes and 
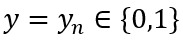
 are the response variable, we model an OSDT tree *d* with a collection of *H* distinct leaves *d* = (*p*_1_,*p*_2_,...,*p_H_*). The objective function in this study integrates the misclassification error with a sparsity penalty imposed on the number of leaf nodes, denoted as *R*(*d*,*x*,*y*). *R*(*d*,*x*,*y*) = *l*(*d*,*x*,*y*) + *λH_d_*, where *l*(*d*,*x*,*y*) represents the misclassification error of the tree, which is computed as the fraction of training data with incorrectly predicted labels. In addition, *H_d_* represents the number of leaves in tree *d*. To regularize the model and discourage larger trees, a regularization term *λH_d_* is introduced, where *λ* is a hyperparameter controlling the strength of the penalty. A higher value of *λ* corresponds to a stronger penalty on the size of the tree. This implies that the tree is more likely to be shallower when achieving optimality.

By using OSDT, we aim to improve the overall performance of the classification task while simultaneously upholding a significant level of interpretability, thereby facilitating a comprehensive understanding of the underlying patterns and factors influencing the classification outcomes.

## Results

### Overview

In this section, we evaluated the proposed method against state-of-the-art machine learning models. We then highlighted attribute importance and provided clear interpretations of derived results within specific patient cohorts for transparency and clarity.

### Performance Evaluation

We demonstrated the performance of our OSDT model by comparing it to commonly used machine learning models as baselines, including naive Bayes, generalized linear model, fast large margin, logistic regression, neural network, vanilla decision tree, random forest, gradient boosted trees, and support vector machine. The evaluation metrics used for the binary classification are accuracy, precision, recall, *F*_1_-score, and *F*_2_-score. Accuracy is a metric that quantifies the overall correctness of a machine learning model. It represented the proportion of correct predictions made by the model across all categories or classes. Precision and recall, on the other hand, measured the model’s ability to accurately predict a specific category or class. Precision focused on the proportion of true positive predictions relative to all positive predictions made by the model. Recall, also known as sensitivity, gauged the model’s capability to correctly detect instances of a specific category. It quantified the proportion of true positive predictions relative to all actual positive instances present in the data. The *F*_1_-score has been widely used in the context of imbalanced classification problems and serves as a prominent metric. It is computed as the harmonic mean of the precision and recall scores, providing a balanced assessment of the model’s performance by considering both precision and recall simultaneously. The *F*_2_-score assigns greater weight to recall than precision, proving beneficial when the consequences of false negatives (ie, missed positive cases where patients are in urgent condition but remain unidentified by the model) outweigh those of false positives (ie, incorrectly identified positive cases). All metrics mentioned exhibited a range of values between 0 and 1, whereby a higher value indicated superior performance.

Compared with various baselines, the performance of the OSDT model achieved the highest accuracy, recall, *F*_1_-score, and *F*_2_-score (Table 2). The performance reported is based on 5-fold cross-validation. These results indicated the predictive capability of the OSDT model in our research context, demonstrating the overall performance and effectiveness of the OSDT model.

**Table 2:** OSDT^a^ performance comparisons with baselines^b^.

Algorithm	Accuracy (%), mean (SD)	Precision (%), mean (SD)	Recall (%), mean (SD)	*F*_1_-score (%), mean (SD)	*F*_2_-score^c^ (%), mean (SD)
Naïve Bayes	78.86 (0.24)	81.3 (7.11)	3.34 (0.59)	6.41 (1.09)	4.13 (1.02)
Generalized linear model	79.23 (0.22)	78.05 (5.00)	5.93 (0.69)	11.01 (1.03)	7.27 (0.93)
Fast large margin	80.26 (0.47)	68.94 (2.57)	17.76 (1.4)	28.21 (1.7)	20.86 (2.17)
Logistic regression	79.26 (0.22)	77.68 (4.26)	6.16 (0.86)	11.41 (1.49)	7.55 (0.78)
Deep learning	80.49 (0.29)	85.59 (4.59)	12.14 (0.39)	21.26 (0.66)	14.66 (0.56)
Decision tree	80.69 (0.2)	69.18 (4.5)	22.45 (4.1)	33.53 (4.5)	25.96 (3.15)
Random forest	79.45 (0.18)	78.19 (5.54)	7.34 (0.31)	13.42 (0.57)	8.96 (2.67)
Gradient boosted trees	80.64 (0.29)	80.8 (2.96)	14.94 (1.55)	25.18 (2.25)	17.85 (1.95)
SVM^d^	80.3 (0.84)	61.42 (5.57)	24.06 (3.4)	34.48 (4.02)	27.39 (1.95)
OSDT (ours)	81.21 (0.20)	68.75 (1.7)	24.56 (0.59)	36.18 (0.66)	28.18 (0.55)

^a^OSDT: optimal sparse decision tree.

^b^OSDT is an algorithm that makes decisions based on direct constraints rather than generating probability scores. As a result, metrics like the receiver operating characteristic curve, precision and recall curve, and area under curve are not applicable for this method.Although the CIs for SVM and OSDT overlap, it is noteworthy that SVM exhibits a significantly larger SD. This indicates that OSDT is more robust in this scenario, delivering a more stable and reliable performance despite the overlapping intervals.

^c^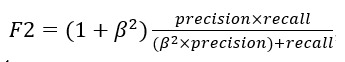; *β*=2.

^d^SVM: support vector machine.

### Interpreting Prediction Results

OSDT, as a tree-based model, possesses the notable advantage of providing interpretable prediction results. We conducted an analysis of the decision trees generated using the entire dataset as well as specific patient cohorts. The objective is to extract the most influential rules that demonstrate both high accuracy and coverage, thereby aiming to uncover the underlying factors that drive the urgent decision of echocardiogram appointments.

We first identified several key categories and attributes that significantly influenced the urgency of patients’ echocardiogram appointments (Table 3). First, the most important categories included “future scheduled process,” pertaining to clinic scheduling policies, and “diagnosis,” indicative of patients’ health conditions. Second, within the top 12 important attributes, a cluster of attributes related to future scheduled processes emerged as the most prominent. These attributes encompassed scenarios if the next downstream appointment following the echocardiogram was scheduled prior to the echocardiogram appointment (ie, “MadeBeforeEcho”), instances where the next appointment did not pertain to the cardiovascular department (ie, “NextDepartment”), cases where no subsequent appointment was scheduled after the echocardiogram appointment (ie, “NextLength_None”), and situations where the time gap between the echo appointment and the subsequent one was less than a day (“NextLength_1”). The absence of a downstream appointment before the echocardiogram could be attributed to the clinic's practice of tailoring subsequent appointments based on the results of the echocardiogram. Consequently, it became imperative for medical providers to accord priority to the echocardiogram appointments of these patients, as the results would furnish vital evidence for guiding appropriate follow-up care and future steps. Third, attributes related to diagnoses assumed the second tier of importance, particularly whether patients exhibited respiratory and cardiac symptoms (ie, “R”) or had documented cardiovascular conditions (ie, “I”). Patients diagnosed with heart-related issues, such as heart murmurs, shortness of breath, and chest pain, typically require expedited access to echocardiography results to determine the next course of action. Fourth, clinical setting attributes and demographic information are also important to patient prioritization. In the context of inpatients, health care providers tended to assign earlier echocardiogram appointment slots as part of a strategy to reduce the length of hospital stays. Additionally, when prioritizing patients with heart conditions, individuals referred by cardiologists received preferential treatment in terms of scheduling. Furthermore, the medical facility providing the data adopted a proactive approach by expediting echocardiogram appointments for out-of-state patients, aiming to minimize their duration of stay. This proactive stance facilitated timely evaluation and management, thereby contributing to a more efficient allocation of resources and an enhanced patient experience. Among medical history attributes, the presence of fluid and electrolyte disorders (ie, “FluidsLytes”) emerged within the top 12, which underscored the strong correlation between fluid and electrolyte disorders and heart failure, further emphasizing its relevance in patient prioritization [29].

These results underscore the significance of admission and policy-related information in determining the urgency of echocardiogram appointments. They reflected the complexities of the scheduling process and highlighted the need for tailored appointment allocation strategies based on patients’ referral status and downstream appointment requirements.

**Figure 2:**
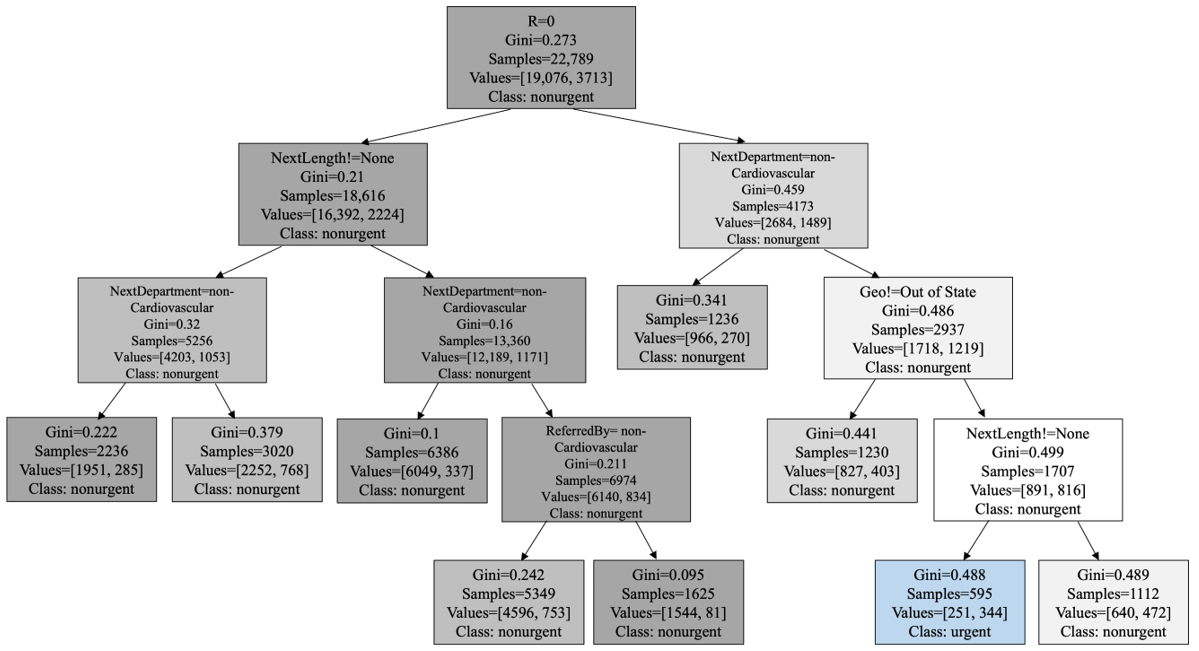
The OSDT for patients whose next downstream appointment after the echocardiogram is scheduled before the date the echocardiogram appointment was generated in the system. OSDT: optimal sparse decision tree. λ=0.0008; accuracy: 83.69%.

We subsequently focus on a specific patient cohort for further analysis. The “MadeBeforeEcho” attribute clearly emerged as exceptionally significant among the dataset’s attributes. It was noteworthy to highlight that, based on the data, there were no urgent cases when the “MadeBeforeEcho” variable was marked as “N.” Consequently, we conducted an investigation specifically focusing on patients whose subsequent downstream appointment was scheduled before the date the echocardiogram appointment was generated in the system. This subset of the patient cohort served as an illustrative example of how decision trees could provide a high degree of interpretability in the context of patient prioritization (Figure 2). Upon scrutiny of the subdecision tree for this cohort depicted, several noteworthy observations emerged. Primarily, it became evident that the most crucial attribute for this cohort is “R,” signifying whether the patient presents with respiratory and cardiac symptoms, which served as the root node of the subtree. The pathway leading to categorizing a patient case as urgent depended on multiple conditions: the patient exhibited respiratory and cardiac symptoms, had an appointment scheduled within the cardiology department, hailed from out of state, and had a subsequent appointment scheduled following the echocardiogram. In contrast, patients without respiratory and cardiac symptoms tended toward classification as nonurgent. This tendency toward nonurgency was particularly pronounced in cases lacking a scheduled appointment subsequent to the echocardiogram.

**Table 3:** Attribute importance and category importance^a^.

Category and attribute			Meanings		Attribute importance
**Future scheduled process (importance=0.0369)**					
	MadeBeforeEcho	Whether the next downstream appointment after echocardiogram is made before the date the echocardiogram appointment was generated in the system or not.		0.0279	
	NextDepartment	The department in which the appointment happened after the date the echocardiogram appointment was generated in the system.		0.0049	
	NextLength_None	No following appointment scheduled after the date the echocardiogram appointment was generated in the system.		0.0035	
	NextLength_1	The number of days from the date the echocardiogram appointment was generated in the system to its following appointment is less than 1 day.		0.0006	
**Diagnoses (importance=0.0154)**					
	R	If have murmur, hypoxemia, shortness, SOB^b^, breath, shock, dyspnea, chest pain, troponin, syncope, electrocardiogram, extremity, mass, swelling, and edema.		0.0147	
	I	If have heart failure, coronary artery, cardiac arrest, STEMI^c^, stroke, cardia, hypertension, endocarditis, NSTEMI^d^, PEA^e^ arrest, AFib^f^, pulmonary embolism, pulmonary hypertension, and vegetation.		0.0007	
**Demographic (importance=0.0369)**					
	Geo_Out of State	Patient is from out of state.		0.0029	
	Geo_Town	Patient is from the local town.		0.0013	
	AGE_19-55	Age between 19 and 55 years.		0.0011	
**Clinical settings (importance=0.0053)**					
	ReferredType	Referred type-inpatient or outpatient.		0.0047	
	ReferredBy_CV	The specialty that patient referred by is cardiovascular disease department.		0.0006	
	FluidsLytes (medical history; importance=0.0021)	If have fluid and electrolyte disorders		0.0021	

^a^The relative importance scores of the attribute category and individual attributes are determined by the Gini index of the optimal sparse decision tree. The feature importance values are relative importance values and do not have a fixed absolute range. We presented only the most important features.

^b^SOB: shortness of breath.

^c^STEMI: ST-elevation myocardial infarction.

^d^NSTEMI: non–ST-elevation myocardial infarction.

^e^PEA: pulseless electrical activity.

^f^AFib: atrial fibrillation.

### Analyses on Diverse Patient Cohorts

In order to enhance the validity of the decision trees and gain more valuable medical insights, we conducted more analyses on smaller patient cohorts. Specifically, we focus on patients who have no next downstream appointment after echocardiogram and are categorized as inpatients. Furthermore, we narrowed down the patient cohort based on specific medical history and presented a compilation of rules extracted from the decision tree (Table 4).

A decision rule was defined as the pathway from the root of a decision tree to a leaf node 
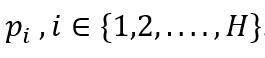
. The accuracy and coverage of a decision rule served as critical metrics for evaluating its effectiveness and applicability. Accuracy, denoting the capacity of a decision rule to effectively forecast the outcome of interest, was quantified as the proportion of records that fulfill both the rule’s precondition and its consequent within the precondition. This metric was computed as 
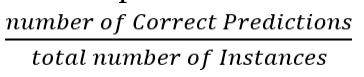
, where “number of Correct Predictions” denoted the count of instances where the decision rule accurately anticipated the desired outcome and “Total number of Instances” represented the entire dataset or the set of instances under consideration, which elucidated how accuracy measures the precision of a decision rule in making predictions based on its specified conditions and its congruence with actual outcomes within the dataset. Coverage, on the other hand, measured the proportion of cases or individuals to which the decision rule could be applied. It could be calculated as 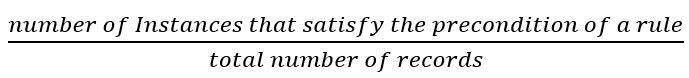. It signified the generalizability and practical scope of the rule in real-world scenarios. A decision rule with high coverage indicates its ability to be applied to a wide range of cases or individuals, thereby increasing its usefulness in practice.

In the context of patients with congestive heart failure (CHF), anemia played a significant role in determining the urgency of echocardiogram appointments (Table 4). Anemia could have detrimental effects on cardiac function through various mechanisms [29]. First, it induces cardiac stress by increasing heart rate and stroke volume. Additionally, anemia could lead to reduced renal blood flow and fluid retention, adding further strain to the heart. Prolonged anemia, regardless of its underlying cause, could contribute to the development of left ventricular hypertrophy, which exacerbates CHF by promoting cardiac cell death through apoptosis. Notably, patients with anemic CHF often exhibited resistance to CHF medications, and numerous studies consistently demonstrated that these individuals have a higher mortality rate compared to patients with non-anemic CHF [30]. Anemia also played a critical role in patients with coagulopathy, as it exacerbated bleeding, which in turn further worsens coagulopathy [30].

For patients with hypothyroidism, fluid and electrolyte disorders served as strong indicators. Hypothyroidism, a prevalent endocrine disorder, was associated with the development of congestive heart failure. Electrolyte disturbances were commonly observed in patients with chronic heart failure [31]. Echocardiogram has been a suitable modality for guiding fluid resuscitation in critically ill individuals. It allowed for the evaluation of fluid responsiveness based on several parameters, such as the left ventricle, aortic outflow, inferior vena cava, and right ventricle [32].

The impact of alcohol consumption on cardiovascular health was multifaceted. Extensive research has demonstrated that the consumption of alcohol at levels surpassing approximately 1 to 2 drinks per day was associated with hypertension [28]. This condition adversely affects the elasticity of arteries, leading to diminished blood and oxygen flow to the heart and consequently contributing to the onset of heart disease [33]. These pathophysiological changes increase the risk of heart disease. Consequently, patients with a history of alcohol abuse and concomitant hypertension might require an urgent echocardiogram to assess the potential cardiac implications arising from these interconnected conditions.

Patients diagnosed with valvular heart conditions would fall into the urgent category if they also exhibited cardiovascular issues and a history of congestive heart failure. These attributes collectively signaled the presence of potentially serious cardiac problems, indicating a compelling need for an echocardiogram to obtain detailed cardiac information and facilitate accurate diagnoses. In the case of patients grappling with depression, their urgency classification as “urgent” was contingent upon the presence of co-occurring health issues. Extensive research has established a substantial influence of depression on the outcomes of concurrent medical conditions. Consequently, when depression coincided with other health problems, it necessitated an “urgent” classification, acknowledging its significant impact on overall health outcomes [34]. Regarding patients with obesity, an “urgent” classification applied if they additionally exhibited fluid and electrolyte disorders. Research findings have illuminated a connection between overweight or obesity and specific physiological factors, such as lower reactance and hypertonicity. Furthermore, individuals with overweight and those with obesity with lower reactance tended to demonstrate significantly elevated serum sodium levels compared to individuals with a normal weight. These associations underscored the importance of promptly addressing the medical needs of patients with obesity with fluid and electrolyte disorders, warranting an “urgent” classification for their cases [35].

Overall, the decision rules extracted from our analyses aligned closely with medical knowledge, providing reliable insights for identifying urgent echocardiogram appointments for patients. The congruence between the rules and medical understanding not only validated the effectiveness of our model but also highlighted the consistent application of medical principles in the decision-making process. This focused analysis contributed to a better understanding of the OSDT model’s validity and offered valuable medical perspectives to enhance the identification of urgent patients’ echocardiogram appointments.

**Table 4:** Decision rules for specific patient cohorts.

Cohort	Rules for a patient to be classified as urgent	Rule accuracy (%)	Rule coverage (%)
CHF^a^	The department in which the appointment happened after the echocardiogram appointment was generated in the system=non-cardiovascular disease, AGE<75, anemia=yes	100	14.20
Coagulopathy	Anemia=Yes	99	53.03
Hypothyroid	Fluid and electrolyte disorders=yes, Whether the patient had a cardiovascular surgery within six months prior to the echocardiogram appointment=no	100	32.91
Alcohol	Hypertension=yes	100	43.75
Valvular	I=1(has cardiovascular conditions), CHF=yes	100	6.36
Depression	Z=1 (has factors influencing health status and contact with health service)	100	24.49
Obesity	Geo!=Town, E=0 (has no nutritional and metabolic diseases), fluid and electrolyte disorders=yes	100	23.75

^a^CHF: congestive heart failure.

## Discussion

### Overview

The primary objective of our study is to forge an effective tree-based classification machine learning model geared toward prioritizing the allocation of echocardiogram appointments for patients with a heightened need for timely diagnostics. Our long-term goal is to streamline the scheduling process, ensuring that patients’ medical requirements are promptly addressed, thereby minimizing delays and optimizing their health care experience. Moreover, our study aspired to delve deeper into the intricate attributes that contribute to the urgency of echocardiogram lab appointments. Recognizing the intricate interplay of medical, logistical, and patient-specific variables, we sought to unravel the complex rules and dynamics that govern appointment prioritization. By harnessing the inherent interpretability of our model, we aim to uncover hidden insights and relationships within a large amount of EHR data, shedding light on the critical determinants that underscore the need for rapid scheduling. The implications of our study extended beyond the realm of predictive modeling. We aimed to empower health care professionals with a powerful tool that not only optimizes resource allocation but also enriches their decision-making process.

### Principal Results

The findings demonstrate promising results by accurately predicting the urgency of echocardiogram appointments and providing valuable insights into the critical guidelines applicable to specific patient cohorts. In summary, the study emphasizes two key points: (1) among the various attributes examined, it is observed that admission-related attributes exert a significant influence on the level of urgency for patients’ echocardiogram appointments; and (2) the urgency of scheduling echocardiogram appointments can be influenced by the presence of comorbidities that exacerbate patients’ conditions. In the case of congestive heart failure, anemia emerges as a significant attribute, highlighting its relevance in contributing to the urgency of echocardiogram appointments. Similarly, coagulopathy is identified as an important attribute for patients with congestive heart failure, further emphasizing the need for prompt assessment. For patients with hypothyroidism, the presence of fluid and electrolyte disorders serves as a concerning indicator, warranting the prioritization of an echocardiogram. Additionally, hypertension is found to be a critical medical knowledge for patients with a history of alcohol abuse, underscoring the urgency of echocardiogram in this population.

Our work is unique in applying an advanced binary decision tree model that offers inherent interpretability, avoiding the limitations of post hoc techniques like local interpretable model-agnostic Explanation and Shapley additive explanation, such as local interpretability constraints, sensitivity to perturbations, and difficulties in selecting appropriate surrogate models. We extract interpretable rules grounded in medical knowledge, making this the first study to introduce tree-based interpretable machine learning for patient prioritization and the stratification of medical test urgency. Furthermore, the tree-based model allows us to derive rules that are easily understandable to medical professionals. These rules can be assessed for alignment with existing medical knowledge and applied in real-world practice by health care providers.

### Limitations

The research has several limitations that could be addressed in future work. First, the accuracy of the prediction model hinges on the quality and completeness of available data; incomplete or missing data may compromise the reliability of predictions. Furthermore, it is essential to recognize that the effectiveness of the model may vary when applied to diverse patient populations or health care settings. This variation can be attributed to the unique attributes and patterns present in the training data, which significantly impact the model’s performance. Moreover, the predictions rely on the elapsed days between the appointment scheduling date and the appointment date. Nonurgent patients may inadvertently be grouped with urgent cases due to cancellations and rescheduling of echocardiogram appointments. While this offers a broad indication of urgency, it may overlook critical factors that influence appointment priority. Integrating essential clinical or contextual details, such as the patient’s medical history, symptom severity, or health care resource availability, into the model could provide more comprehensive insights.

### Conclusions

This research adapts the OSDT algorithm to assess the urgency of patients in need of echocardiograms. The OSDT model demonstrates better performance over alternative machine learning models, highlighting its predictive accuracy and effectiveness. Furthermore, it identifies key attributes and rules governing the prioritization of echocardiogram appointments.

The analysis of decision trees generated by the OSDT model reveals the significance of admission- and policy-related attributes, such as downstream appointment scheduling and patient referral status, in determining appointment urgency. Moreover, the analyses of specific patient cohorts provide medical insights into the role of comorbidities, such as anemia in patients with CHF and coagulopathy, and fluid and electrolyte disorders in patients with hypothyroidism. These insights align with established medical knowledge and enhance the identification of urgent echocardiogram appointments.

In summary, this study facilitates the development of effective scheduling protocols for echocardiogram appointments by harnessing machine learning techniques and integrating medical insights. This approach enhances the overall efficiency and effectiveness of echocardiogram services, ultimately benefiting patient care. The findings can also be generalized to inform the establishment of efficient scheduling protocols and the promotion of equitable access to various other medical laboratory tests.

## Abbreviations

CHF: congestive heart failure
EHR: electronic health record
OSDT: optimal sparse decision tree
